# Exact, time‐dependent analytical equations for spiral trajectories and matching gradient and density‐correction waveforms

**DOI:** 10.1002/mrm.70053

**Published:** 2025-09-13

**Authors:** Guruprasad Krishnamoorthy, James G. Pipe

**Affiliations:** ^1^ Department of Radiology University of Wisconsin Madison Wisconsin USA

**Keywords:** spiral gradient, k‐space trajectory design

## Abstract

**Purpose:**

To analytically define a spiral waveform and trajectory that match the constraints of gradient frequency, slew rate, and amplitude.

**Theory and Methods:**

Piecewise analytical solutions for gradient waveforms under the desired constraints are derived using the circle of an involute rather than an Archimedean spiral. Also given are the analytical equations for the time‐dependent k‐space trajectory and sampling density compensation weights, and analytical expressions for the time dependence of data acquisition in k‐space. Open‐source software implementing all these equations is shared. Performance is measured against numerically derived solutions to an Archimedean spiral. Scanner implementation is illustrated.

**Results:**

The performance of the proposed equations is very similar to that of numerically derived solutions, but this method is much easier to implement and analyze.

**Conclusion:**

The proposed method, WHIRLED PEAS (Winding Hybrid Interleaved Radial Lines Encoding Described by Piecewise Exact Analytical Solution), is an easy‐to‐implement solution for spiral MRI that performs comparable to optimal numerical designs.

## 1 INTRODUCTION

The use of spiral k‐space trajectories for data acquisition in MRI^1^, ^2^ is well established, with the benefits of high SNR and temporal efficiency, short minimum TEs, as well as reduced and incoherent artifacts from some types of motion. These trajectories traditionally follow an Archimedean spiral under some constraints for gradients (e.g., maximum gradient amplitude or slew rate). Two approaches to deriving optimal gradient waveforms that sample along this Archimedean spiral include approximately optimal analytical solutions^3^, ^4^, ^5^ and numerical methods.^6^, ^7^ These references reflect only a small portion of the methods published to date; however, to the authors' knowledge, there have been no exact analytical solutions, particularly none that allow gradient frequency limitations or include analytical solutions for both gradient waveforms and k‐space trajectories.

A variant of the spiral trajectory, which follows the involute of a circle, was previously introduced with the acronym WHIRL (Winding Hybrid Interleaved Radial Lines).^8^ This trajectory is similar to an Archimedean spiral, with two exceptions: (1) it forces the sampling normal to the trajectory (e.g., arm‐to‐arm spacing) to stay exactly uniform; and (2) it is not defined within a prescribed central circle. The first property makes it slightly more optimal than an Archimedean spiral purely in terms of path length.^9^ In contrast, the second property requires that one finds a bridging trajectory between the center of the measurement plane (k‐space origin) and the edge of the circle where the trajectory becomes defined. Notably, the slight difference in the math describing this trajectory allows it to be analytically defined under various gradient optimization constraints such as frequency, slew, and gradient magnitude. The inclusion of frequency is particularly relevant because the fidelity of gradient waveforms is known to depend on their frequency content.^10^ The proposed trajectory is completed by adding a quarter‐circle arc at the beginning of the trajectory to span the inner circle to the k‐space origin and a quarter‐circle arc at the end to bend the trajectory into a completely angular direction. The first arc is necessary, and the second arc makes the trajectory highly modular, as will be shown.

Expanding on previously described work dubbed *WHIRL Encoding Described by Piecewise Exact Analytical Solutions* (WHIRLED PEAS),^11^, ^12^ the authors give exact, optimal analytic expressions for the gradient waveform, k‐space trajectory, and weights for sampling density correction (SDC). This work also gives a map of acquisition time as a function of k‐space, which is useful for deblurring algorithms and equations for predicting (or enforcing) the duration of a spiral waveform. Additional logic is presented to create optimal trapezoids for zeroth and first‐moment nulling of spiral (and other rotated) gradient waveforms. The method was implemented on a scanner, and example images and waveforms are given to illustrate its performance and utility. Code is provided for calculations required for all gradient waveforms, trajectories, weights, time maps, and trapezoids mentioned above.

## 2 THEORY

**FIGURE 1:**
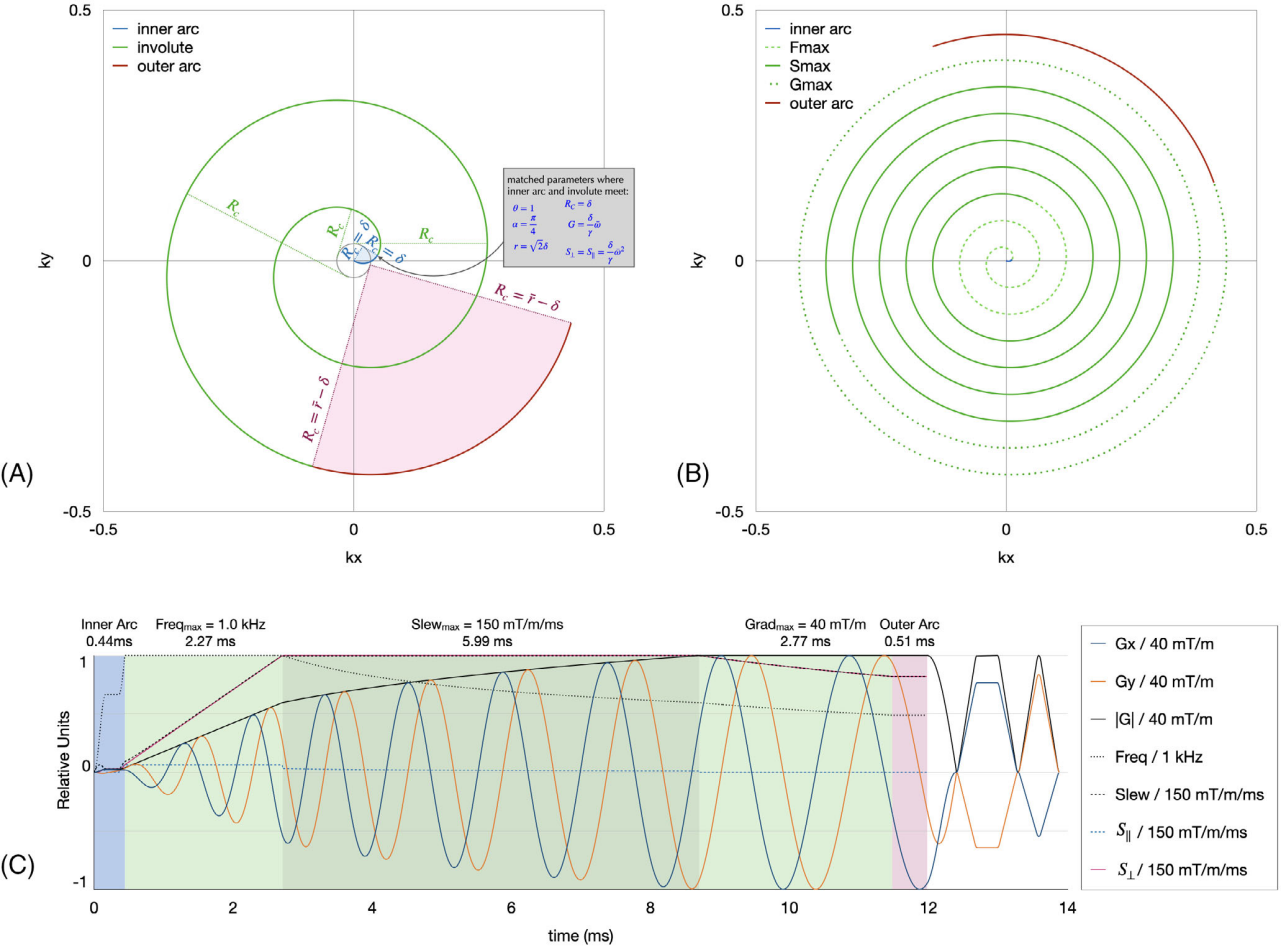
Examples of (A,B) WHIRLED PEAS k‐space trajectories and (C) gradient waveforms. A short‐readout trajectory (A) shows the three components of the trajectory and the changing radius of curvature *R*_c_: The inner arc (blue), tracing 90° *with Rc = delta rotating about a single point, followed by (B) the involute of an inner (black) circle of radius delta, with the trajectory (green) following the path of a string unwrapped from that circle, and ending with (C) an outer arc (red) of fixed Rc rotating about a single point through 90°. R*c, radius of curvature; WHIRLED PEAS, Winding Hybrid Interleaved Radial Lines Encoding Described by Piecewise Exact Analytical Solutions.

Illustrative k‐space trajectory and gradient waveforms are shown in Figure 1 for reference in the rest of the theory section. This section outlines the steps needed in order to jointly solve for the k‐space trajectory and gradient as a function of time.

### 2.1 Circle involute basics

The main part of the WHIRL trajectory is the involute of a circle of radius δ, describing the path of the end of a string being unwrapped from that circle. One can visualize this trajectory as the green paths in Figure 1A, B. Note in Figure 1A that the line *R*_C_, which is always tangential to the central circle, has a length equal to the amount of “string” unwrapped around that circle. This also gives the trajectory's radius of curvature and has length

(1) $$R_{C}=\sqrt{r^{2}-\delta^{2}}=\delta \;\theta .$$

Starting the *N* trajectories at the perimeter of that circle so that they are the Nyquist distance apart for some FOV F requires

(2) $$\delta =\frac{N}{2\pi F}.$$

### 2.2 Coupling the gradient and k‐space trajectories

When imaging in the {*x*, *y*} plane, one can write the gradient *g* and k‐space trajectory *k* as complex variables, that is,

(3) $$g=g_{x}+i\;g_{y}=Ge^{i\theta },\text{and}$$

(4) $$k=k_{x}+i\;{\mathrm{kg}}_{y}=re^{\mathrm{i\phi}}.$$

The time‐dependent variables (*G*, *r*) and (θ, ϕ) are the magnitudes and phases of *g* and k, respectively; the temporal dependence of these four primary variables are jointly solved in this work.

Given the angle α of the gradient trajectory away from the radial direction,

(5) α=θ−ϕ,

the WHIRL spiral is defined^8^ outside of δ purely by enforcing

(6) $$\cos\left(\alpha \right)=\frac{\delta }{r}.$$

Equation (6) allows one to write the progression through k‐space due to the gradient amplitude *G* as time derivatives of *r* and ϕ, that is,

(7) $$r^{\prime }=\gamma G\;\cos\left(\alpha \right)=\gamma G\;\frac{\delta }{r},\text{and}$$

(8) $$\phi^{\prime }=\frac{\gamma G\;\sin\left(\alpha \right)}{r}=\frac{\gamma G\;\sqrt{r^{2}-\delta^{2}}}{r^{2}},$$

where γ is the gyromagnetic ratio. A key relationship between variables can be found using Equations ((5), (6), (7), (8))–((5), (6), (7), (8)) to simplify the time derivative of θ, giving

(9) $$\theta^{\prime }=\alpha^{\prime }+\phi^{\prime }=\frac{\gamma G}{R_{c}}.$$

The second equality in Equation (9) comes after some trigonometric and algebraic simplification. This is not possible when using a conventional Archimedean spiral, which enforces^6^ tan(α)=(r/δ). Finally, taking the derivative of Equation (3) shows that the gradient slew rate magnitude is

(10) $$\left|\frac{\mathrm{dg}}{\mathrm{dt}}\right|=\sqrt{{\left(G^{\prime }\right)}^{2}+{\left(G\;\theta^{\prime }\right)}^{2}}=\sqrt{{S_{∥}}^{2}+{S_{⊥}}^{2},}$$

where $S_{∥}$ and $S_{⊥}$ are defined as the parts of the slew vector parallel and orthogonal to the gradient direction, respectively. Later analysis will show that $S_{⊥}$ is a very good approximation to the slew magnitude for most of the trajectory; this is adopted here, which allows Equation (9) to be extended as

(11) $$\theta^{\prime }=\frac{\gamma G}{R_{c}}=\frac{S_{⊥}}{G}.$$

### 2.3 Solutions under three constraints

Importantly, WHIRL (Equation (6)) is not defined for *r*<δ, for which the N arms must be closer than the Nyquist limit. Outside this region, the WHIRL trajectory can be solved, using Equations ((1), (2), (3), (4), (5), (6), (7), (8), (9), (10), (11))–((1), (2), (3), (4), (5), (6), (7), (8), (9), (10), (11)), in three segments. These segments correspond to constraints from a maximum frequency $\bar{f}$ (or angular frequency $\bar{\omega }=2\pi \bar{f}$), a maximum orthogonal slew rate ${\bar{S}}_{⊥}$, and a maximum gradient amplitude $\bar{G}$, which ends when one nears the maximum desired k‐space radius $\bar{r}$, as seen in Figure 1B, C. The solutions, respectively, are:

Constraint 1: fixed maximum gradient frequency $\bar{f}$ (or angular frequency $\bar{\omega }=2\pi \bar{f}$)

(12a) $$\theta =\bar{\omega }\;t$$

(12b) $$G=\frac{R_{c}}{\gamma }\bar{\omega }=\frac{\delta }{\gamma }{\bar{\omega }}^{2}t$$

Constraint 2: fixed maximum slew rate $S_{⊥}=\bar{S}$

(13a) $$\theta =\sqrt[{3}]{\frac{9\gamma \bar{S}}{4\delta }}\sqrt[{3}]{t^{2}}$$

(13b) $$G=\sqrt[{3}]{\frac{3\delta {\bar{S}}^{2}}{2\gamma }}\sqrt[{3}]{t}$$

Constraint 3: fixed maximum gradient amplitude $G=\bar{G}$

(14a) $$\theta =\sqrt{\frac{2\gamma \bar{G}}{\delta }}t$$

(14b) $$G=\bar{G}$$

In all three cases,

(15) $$\phi =\theta -\arccos\left(\frac{\delta }{r}\right)$$

and

(16) $$r=\delta \sqrt{\theta^{2}+1}.$$

### 2.4 Inner arc

To define the spiral trajectory for *r* < δ, a quarter‐circle k‐space arc of radius δ is used to connect the center of k‐space to the circle involute described above, as illustrated in Figure 1A–C in blue. This inner arc, which itself has a fixed radius of curvature *R*_C_ = δ, is designed to meet the WHIRL trajectory at a k‐space radius of *r* = $\sqrt{2}\delta$, and θ=1. Knowing the relevant variables for the WHIRL trajectory at this point allows one to create an appropriate gradient waveform that matches the gradient, slew, and k‐space waveforms smoothly between the arc and the WHIRL trajectory. There are many gradient solutions to achieve this inner‐arc trajectory; for this work the arc is defined in three stages played sequentially,

(17) $$\begin{array}{l} \mathrm{arc}.a:S_{∥}=C*\sin\left(\omega t\right),0<t<\mathrm{TA}\\ \mathrm{arc}.b:S_{∥}=0,0<t<\mathrm{TB}\\ \mathrm{arc}.c:S_{∥}=C*\sin\left(\omega t\right),0<t<\frac{\mathrm{TA}}{2}. \end{array}$$

From Equation (10), *G* is the integral of $S_{∥}$, θ is defined to maintain the circular trajectory on the arc, and the variables *C*, ω, t are local to the stage, and TA and TB are chosen to smoothly match the WHIRL trajectory at the point of intersection, including all the parameters shown in the gray inset of Figure 1A. This smooth inner arc trajectory mitigates gradient and slew rate performance requirements near the k‐space center to increase trajectory fidelity; however, the total duration of this arc is typically a few hundred μs.

### 2.5 Outer arc

We have extended the previous WHIRLED PEAS to include a quarter‐circle k‐space arc at the outer edge of the involute as well, such that the trajectory ends in a purely angular direction (α = 0), as seen in red in Figure 1A–C. This outer arc with a radius of curvature ${\bar{R}}_{C}=\bar{r}-\delta$ joins smoothly with the outer edge of the involute and allows mirror images of the trajectory to be pieced together for seamless spiral out/spiral in acquisitions with no additional trajectory calculations. The addition of this outer arc increases the required readout time τ by a few microseconds and decreases the total gradient time by a few microseconds due to a slight reduction in required refocusing areas; neither change is substantial.

### 2.6 Solved equations

The complete WHIRLED PEAS equations for the inner and outer arcs along with the circle involute under the three constraints are given in Table 1. They are written as sections that are played sequentially, with the relative time variable t in each section going from *T*_start_ to *T*_end_; these were calculated to smoothly match gradient, k‐space between each segment. In each section there are analytical equations for both the gradient and k‐space vectors, along with the SDC weights, which allows each to be directly determined with its own dwell time. The SDC weights were derived^2^ as the product of G (from Table 1) and the arm spacing, which is 1 for the entire involute section and sin(θ) for the arc section, using θ as given in Table 1. When *T*_end_ < *T*_start_ for any WHIRL section, this signifies that this section will be skipped; this is easily implemented by lowering the relevant constraint to make *T*_end_ = *T*_start_.

**TABLE 1:** WHIRLED PEAS equations for gradient waveform, k‐space trajectory, and SDC weights.

Stage	*T*_start_	*T*_end_	*G*	θ (see note a)	*r*	ϕ ^a^(see note a)	SDC
Inner arc part a	0	$\left[\frac{\pi }{3\bar{\omega }}\right]$	$\frac{\delta \bar{\omega }}{3\gamma }\left(1-\cos\left(3\bar{\omega }t\right)\right)$	$\frac{\bar{\omega }t}{3}-\frac{\sin\left(3\bar{\omega }t\right)}{9}$	$2\delta \sin\left(\frac{\theta }{2}\right)$	$\mathrm{ta}n^{-1}\left[\frac{1-\cos\left(\theta \right)}{\sin\left(\theta \right)}\right]$	$\frac{G}{\bar{G}}\sin\left(\theta \right)$
Inner arc part b	0	$\frac{2\pi +1}{6\bar{\omega }}$	$\frac{2\delta \bar{\omega }}{3\gamma }$	$\frac{\pi }{9}+\frac{2\bar{\omega }t}{3}$			
Inner arc part c	0	$\frac{\pi }{6\bar{\omega }}$	$\frac{\delta \bar{\omega }}{3\gamma }\left[3-\cos\left(3\bar{\omega }t\right)\right]$	$\frac{1+3\pi +9\bar{\omega }t-\sin\left(3\bar{\omega }t\right)}{9}$			
$\bar{\omega }$	$\frac{1}{\bar{\omega }}$	$\frac{\gamma {\bar{S}}_{⊥}}{\delta {\bar{\omega }}^{3}}$	$\frac{\delta }{\gamma }{\bar{\omega }}^{2}t$	$\bar{\omega }t$	$\delta \sqrt{\theta^{2}+1}$	$\theta -\text{acos}\left(\frac{\delta }{r}\right)$	$\frac{G}{\bar{G}}$
${\bar{S}}_{⊥}$	$\frac{2\gamma {\bar{S}}_{⊥}}{3\delta {\bar{\omega }}^{3}}$	$\frac{2\gamma {\bar{G}}^{3}}{3\delta {\bar{S}}_{⊥}^{2}}$	$\sqrt[{3}]{\frac{3\delta {\bar{S}}_{⊥}^{2}}{2\gamma }}t^{1/3}$	$\sqrt[{3}]{\frac{9\gamma {\bar{S}}_{⊥}}{4\delta }}\;t^{2/3}$			
$\bar{G}$ (see note b)	$\frac{\gamma {\bar{G}}^{3}}{6\delta {\bar{S}}_{⊥}^{2}}$	$\frac{{\bar{R}}_{c}^{2}}{2\gamma \delta \bar{G}}$	$\bar{G}$	$\sqrt{\frac{2\gamma \bar{G}}{\delta }}t^{1/2}$			
Outer arc (see note b)	0	$\frac{\pi {\bar{R}}_{c}}{2\gamma \bar{G}}$	$\bar{G}$	$\frac{{\bar{R}}_{c}}{\delta }+\frac{\gamma \bar{G}}{{\bar{R}}_{c}}t$	(see note c)	(see note d)	$\cos\left(\frac{\gamma \bar{G}}{{\bar{R}}_{c}}t\right)$

*Note*: For each trajectory stage, the variable t is local to that stage and varies from *T*_start_ to *T*_end_. The duration of the stage is therefore to *T*_end_ − *T*_start_. When connecting stages, t is reset for each piece rather than being a single continuous time variable across all stages.

a. For implementation, the inner arc (both θ and ϕ) is rotated by ($1-\frac{\pi }{2}$) radians to match the involute trajectory.

b. ${\bar{R}}_{c}=\bar{r}-\delta .$

c. $r=\sqrt{\delta^{2}+{\bar{R}}_{c}^{2}+2\delta {\bar{R}}_{c}\sin\left(\frac{\gamma \bar{G}}{{\bar{R}}_{c}}t\right)}.$

d. $\phi =\frac{{\bar{R}}_{c}}{\delta }-\cos^{-1}\left(\frac{\delta +{\bar{R}}_{c}\sin\left(\frac{\gamma \bar{G}}{{\bar{R}}_{c}}t\right)}{r}\right).$

Abbreviations: *R*c, radius of curvature; SDC, sampling density correction; WHIRLED PEAS, Winding Hybrid Interleaved Radial Lines Encoding Described by Piecewise Exact Analytical Solutions.

Summing up the total times for each section (*T*_start_–*T*_end_) gives the total acquisition time τ, that is,

(18) $$\tau =\frac{5\left(\pi -1\right)}{6\bar{\omega }}+\frac{\pi {\bar{R}}_{c}}{2\gamma \bar{G}}+\frac{2\pi F}{N}\left[\frac{\gamma {\bar{S}}_{⊥}}{3{\bar{\omega }}^{3}}+\frac{\gamma {\bar{G}}^{3}}{6{\bar{S}}_{⊥}^{2}}+\frac{{\bar{R}}_{C}^{2}}{2\gamma \bar{G}}\right],$$

where ${\bar{R}}_{C}=\sqrt{\bar{r^{2}}-\delta^{2}}$. One can also use this to calculate *N* in order to achieve a desired τ. Because δ, and therefore ${\bar{R}}_{c}$, depend on *N*, a more exact solution can be arrived at iteratively.

With these k‐space time‐dependent equations, one can create analytical equations for the time of acquisition for any point in k‐space. This is done for the WHIRL trajectory by inverting the equations for θ in Table 1 and using Equation (1) to write the acquisition time as a function of *R*_C_. For the initial arc section, which is quite small, a simple linear approximation of time as a function of r was derived, which is accurate with tens of microseconds. These equations are shown in Table 2. This time dependence is useful to describe the corruption of k‐space data by off‐resonance induced phase, which creates blurring in the image. Our group uses this time description, along with a collected off‐resonance map, for a forward blurring model used in an iterative deblurring procedure.^13^

**TABLE 2:** WHIRLED PEAS equations for approximate time of acquisition in k‐space.

Stage	*r*_start_ or *R*_C start_	*r*_end_ or *R*_C end_	*t*_base_	*t*(r) *or t*(*R*_C_) (add *t*_base_ for time)
arc (r)	0	$r=\sqrt{2}\delta$	0	$\approx \frac{\pi }{6\bar{\omega }}+\frac{4\pi +1}{6\sqrt{2}\delta \bar{\omega }}r$
$\bar{\omega }$ (R_C_)	δ	$\frac{\gamma {\bar{S}}_{⊥}}{{\bar{\omega }}^{2}}$	$\frac{5\left(\pi -1\right)}{6\bar{\omega }}$	$\frac{R_{C}}{\delta \bar{\omega }}$
${\bar{S}}_{⊥}$ (*R*_C_)	$\frac{\gamma {\bar{S}}_{⊥}}{{\bar{\omega }}^{2}}$	$\frac{\gamma {\bar{G}}^{2}}{{\bar{S}}_{⊥}}$	$\frac{5\left(\pi -1\right)}{6\bar{\omega }}+\frac{\gamma {\bar{S}}_{⊥}}{3\delta {\bar{\omega }}^{3}}$	$\frac{2}{3\delta }\sqrt{\frac{R_{C}^{3}}{\gamma {\bar{S}}_{⊥}}}$
$\bar{G}$ (*R*_C_)	$\frac{\gamma {\bar{G}}^{2}}{{\bar{S}}_{⊥}}$	${\bar{R}}_{C}=\sqrt{{\bar{r}}^{2}-\delta^{2}}$	$\frac{5\left(\pi -1\right)}{6\bar{\omega }}+\frac{\gamma {\bar{S}}_{⊥}}{3\delta {\bar{\omega }}^{3}}+\frac{\gamma {\bar{G}}^{3}}{6\delta {\bar{S}}_{⊥}^{2}}$	$\frac{R_{C}^{2}}{2\gamma \delta \bar{G}}$

### 2.7 Moment nulling trapezoids

**FIGURE 2:**
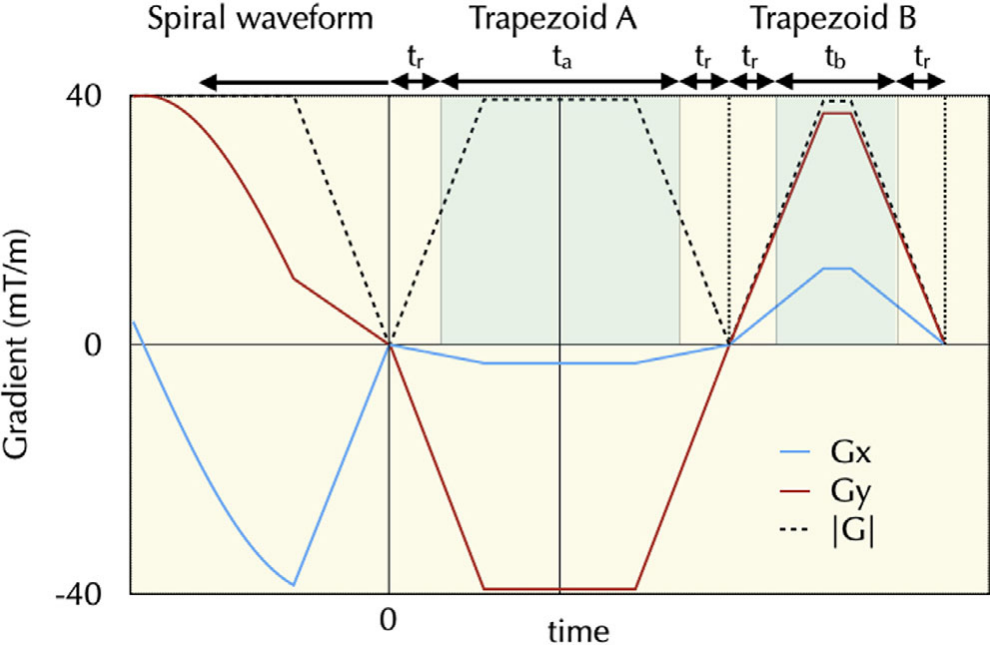
Illustration of trapezoids used for pair‐wise design of M1 and M0 nulling with full rotational compatibility.

Designing a pair of trapezoids to null zeroth and first moments of a single gradient waveform is common; for spiral imaging, the design of “vector” trapezoid pairs that are played out in both in‐plane directions and are freely rotatable under gradient constraints has not been shown to our knowledge. We have developed a fast algorithm for joint design of this trapezoid pair, which reduces their total duration compared with those made using separate, single‐channel designs. Joint design ensures simultaneous nulling across both axes while maximizing gradient usage without exceeding system limits, resulting in more efficient and symmetric gradient waveforms. Using the notation illustrated in Figure 2, we define variables *t*_*a*_ and *t*_*b*_ to be the effective total duration (area divided by amplitude) of two trapezoids A and B, respectively, and the effective ramp time *t*_*r*_ to be one‐half the actual ramp time. Defining M0 and M1 as the relative gradient moments at the end of spiral waveforms, we write matrix equations for the desired moments of the gradient waveforms and invert them, giving

(19) $$\left[\begin{array}{l} g_{\mathrm{xa}}\\ g_{\mathrm{xb}} \end{array}\right]=\frac{\left[\begin{array}{ll} t_{b}\left(2t_{\mathrm{ra}}+t_{\mathrm{rb}}+t_{a}+0.5t_{b}\right) & -t_{b}\\ -\;t_{a}\left(t_{\mathrm{ra}}+0.5t_{a}\right) & t_{a} \end{array}\right]}{t_{a}t_{b}\left(t_{\mathrm{ra}}+t_{\mathrm{rb}}+0.5\left(t_{a}+t_{b}\right)\right)}\left[\begin{array}{l} -\;M0_{x}\\ -\;M1_{x} \end{array}\right]$$

(20) $$\left[\begin{array}{l} g_{\mathrm{ya}}\\ g_{\mathrm{yb}} \end{array}\right]=\frac{\left[\begin{array}{ll} t_{b}\left(2t_{\mathrm{ra}}+t_{\mathrm{rb}}+t_{a}+0.5t_{b}\right) & -t_{b}\\ -\;t_{a}\left(t_{\mathrm{ra}}+0.5t_{a}\right) & t_{a} \end{array}\right]}{t_{a}t_{b}\left(t_{\mathrm{ra}}+t_{\mathrm{rb}}+0.5\left(t_{a}+t_{b}\right)\right)}\left[\begin{array}{l} -\;M0_{y}\\ -\;M1_{y} \end{array}\right]$$

(21) $$\left[\begin{array}{l} t_{a}\\ t_{b} \end{array}\right]=\frac{\left[\begin{array}{ll} g_{\mathrm{yb}} & -g_{\mathrm{xb}}\\ -\;g_{\mathrm{ya}} & g_{\mathrm{xa}} \end{array}\right]}{\left(g_{\mathrm{xa}}g_{\mathrm{yb}}-g_{\mathrm{xb}}g_{\mathrm{ya}}\right)}\left[\begin{array}{l} -\;M0_{x}\\ -\;M0_{y} \end{array}\right].$$

We enforce these equations in the following iterative solution:

Step 0: Initialize with *t*_*ra*_ = *t*_*rb*_ = 0.5*Gmax/Smax, *t*_*a*_ = 2 *t*_*ra*_, *t*_*b*_ = *t*_*rb*_.

Step 1: Solve for gradient amplitudes, using Equations (19) and (20).

Step 2: Normalize A and B vector gradient amplitudes separately by factors $\frac{g_{\max}}{\sqrt{g_{\mathrm{xa}}^{2}+g_{\mathrm{ya}}^{2}}}$ and $\frac{g_{\max}}{\sqrt{g_{\mathrm{xb}}^{2}+g_{\mathrm{yb}}^{2}}}$, respectively.

Step 3: Solve for new trapezoid durations, using Equation (21).

Iterating these 3 steps converges on a solution quickly. One can modify this approach routine (see code) to account for nontrapezoid (triangular) waveforms.

## 3 METHODS

### 3.1 Timing and relative SNR comparison

The total sampling time τ for WHIRLED PEAS was compared to that of a numerical method^7^ for achieving optimal gradient waveforms for an Archimedean trajectory, for a variety of use cases. For the numerical Archimedean trajectory, both the layered conditioning that smooths the waveforms across segments and the implemented magnetisation transfer function (MTF)‐informed constraints were turned off, to be consistent with WHIRLED PEAS, which does not have these features. The numerical trajectory did have a “starting window” of 200 μs in which the slew rate ramped up the maximum slew, to be consistent with the gentler start of the WHIRLED PEAS trajectory. Also, although the numerical trajectory was limited by the total slew, the same number used for WHIRLED PEAS constraint limited only $S_{⊥}$. This was a very minor difference, as shown below; however, it did slightly advantage the WHIRLED PEAS trajectory.

The pixel SNR loss W due to SDC weighting, given by

(22) $$W=\frac{\sum w_{n}}{\sqrt{N\sum w_{n}^{2}}},$$

was also computed for both trajectory designs for the same use cases. Here, $w_{n}$ is the density weight applied to the *n*th sample in a spiral readout with *N* as the total number of readout samples. For the numerical trajectory, SDC was calculated numerically,^14^ whereas the WHIRLED PEAS SDC was calculated from Table 1.

### 3.2 Slew constraint

The slew constraint for WHIRLED PEAS is defined only by $S_{⊥}=G\theta^{\prime }$ and does not include $S_{∥}=G^{\prime }$, where the total slew is $S=\sqrt{S_{⊥}^{2}+S_{∥}^{2}}$. This was necessary for deriving an analytical solution but could hypothetically present complications because (1) PNS and scanner hardware limits depend on S, not $S_{⊥}$; and (2) there are discontinuities in both $S_{∥}$ and *S* immediately before and after the ${\bar{S}}_{⊥}$‐constrained segment. As illustrated in Figure 1C, these are small but more pronounced in $S_{∥}$, and very small in *S*. These discontinuities in slew‐add frequency components to the gradient waveform that may result in undesirable spikes in a precompensated waveform and also degrade trajectory fidelity. The largest value of $S_{∥}$ and *S*, as well as the largest change in these two values, happens at the end of the $\bar{\omega }$‐constrained segment, where $S_{⊥}={\bar{S}}_{⊥}$ and

(23) $$S_{∥}=\frac{\delta {\bar{\omega }}^{2}}{\gamma },$$

while at the start of the ${\bar{S}}_{⊥}$‐constrained segment, $S_{⊥}={\bar{S}}_{⊥}$ and

(24) $$S_{∥}=\frac{\delta {\bar{\omega }}^{2}}{2\gamma }.$$

To determine the extent of this disparity, the slew value $S_{∥}$ from Equation (23), and the total slew *S* at that point (which is the greatest slew during the waveform), were calculated for various use cases.

### 3.3 Software and scanner implementation

The proposed algorithm for generating gradient waveforms, k‐space coordinates, and sampling density compensation weights was implemented as a graphical programming interface (GPI)^15^ node using a combination of C++ and Python. The implementation consists of multiple modules responsible for different aspects of spiral trajectory generation and processing. The implementation includes a GPI node (WHIRLEDPEAS_GPI.py) for simulation and testing, utilizing PyBind11 to call C++ functions from Python, which serves as an interactive environment for modifying parameters and visualizing the generated trajectories before scanner execution. The core functionality of the software implementation is divided into three main components:

1. Base spiral generation (WPGen.cpp)
  The WPGen C++ class generates the base spiral‐out waveform and the M0 and M1 moment nulling trapezoids based on user‐defined constraints such as FOV, resolution, readout duration (τ) or number of interleaves (N), gradient, and slew rate limitations. Smooth trapezoidal transitions are incorporated to minimize high‐frequency content, improving gradient system compatibility while preserving the M0 and M1 moment nulling. If the user prescribes τ, N was computed with,

$$\tau_{\text{rings}}=\left(\frac{5\left(\pi -1\right)}{6\bar{\omega }}+\frac{\pi \bar{r}}{2\gamma \bar{G}}\right),$$

and

(25) $$N=\frac{2\pi F}{\left(\tau -\tau_{\text{rings}}\right)}\left\lfloor \frac{\gamma {\bar{S}}_{⊥}}{3{\bar{\omega }}^{3}}+\frac{\gamma {\bar{G}}^{3}}{6{\bar{S}}^{2}}+\frac{{\bar{r}}^{2}}{2\gamma \bar{G}}\right\rfloor .$$

2 Waveform composition (WPCompose.cpp)
  The WPCompose C++ class takes one or more WPGen objects as inputs and flips, stitches, and arranges them to construct the final k‐space trajectory. It supports four trajectory types: spiral‐out, spiral‐in (by flipping about an axis), spiral in‐out (concatenated spiral‐in and out sections), and spiral out‐in (inverse of spiral in‐out). The flipping operation that is performed at about an angle phi at the end of the second arc, as given in Table 1 is always $\bar{\phi }=\frac{{\bar{R}}_{c}}{\delta }$. For multi‐echo acquisitions, the M1 moment cancels out naturally for the second echo, simplifying implementation.
3 Spiral ordering (SpiralOrder.cpp)
  The SpiralOrder module generates interleaved ordering angles for multi‐shot acquisitions. It supports various arm ordering strategies, including linear, skip, two‐way, and mixed. as described in Ref. 16.

The complete software implementation, as archived at the time of publication—including the GPI node for simulation and the C++ modules—is available in WHIRLEDPEAS.zip.

Additionally, the WHIRLEDPEAS node will be included in GPI Core Node Library version 2.3.3 and later. It can be installed via Conda from the Conda‐Forge channel or cloned as source code from: SourceCodeAtGithub.

For scanner‐side deployment, the C++ modules were used directly without Python dependencies. This enabled an interactive scanner interface, allowing tuning of parameters such as τ, gradient amplitude, slew rate, resolution, and FOV. Full k‐space trajectories and SDC weights are precomputed during protocol setup and passed directly to the reconstruction pipeline. Additionally, the k‐space coordinates are used in real time to apply prospective FOV off‐center shift correction, avoiding unnecessary oversampling and improving spatial accuracy.

To evaluate performance on the scanner, three sequences were developed:

1. A 2D spin‐echosequence.
2. A 3D single‐echo gradient‐echo sequence.
3. A 3D multi‐echo gradient‐echo sequence.

All in vivo imaging was performed on a 3 T GE Signa Premier scanner (GE Healthcare, Waukesha, WI, USA) using a 16‐channel head coil, in compliance with institutional review board guidelines and after obtaining written informed consent from a healthy volunteer. The scan parameters for these sequences are provided in Table 3. Reconstruction of spiral‐in and spiral‐out data was performed independently, gridding, deblurring, and finally, combination in the complex image space for the reconstructed images. Off‐resonance maps for the deblurring process were collected in a prescan using a multi‐echo multi‐shot gradient echo Cartesian sequence. All reconstruction steps were executed using GPI.

## 4 RESULTS

### 4.1 Timing and relative SNR comparison

The parameters for the different tested use cases, as well as the slew information, trajectory duration t, and relative SNR W, are shown in Table 4. These use cases illustrate that the WHIRLED PEAS algorithm produces results very similar to numerical results despite requiring far less computation (for each time point, WHIRLED PEAS requires just two computations from Table 2, whereas the numerical software outlined in Pipe and Borup^7^ has 170 lines of code per time point) and giving an array of analytical expressions for characterization and implementation.

**TABLE 4:** Use case comparisons between WHIRLED PEAS, a numerical Archimedean method.

							WHIRLED PEAS				Numerical	
Use case	$\bar{G}$ (mT/m)	$\bar{S}$ or ${\bar{S}}_{⊥}$ (mT/m/ms)	$\bar{f}$ (kHz)	FOV (cm)	Res (mm)	N Arms	$S_{∥}$ (mT/m/ms)	S = $\;{\left(S_{⊥}^{2}+S_{∥}^{2}\right)}^{1/2}$	τ (ms)	W Equation (10)	τ (ms)	W Equation (10)
1	30	120	1	36	1.2	16	6.56	120.18	15.37	0.940	15.41	0.934
2	40	150	1.2	24	0.7	12	10.62	150.38	27.36	0.968	27.35	0.964
3	50	150	2	20	2	1	2.95	150.03	51.16	0.955	50.58	0.946
4	40	150	1	40	2	10	3.69	150.05	12.37	0.886	12.42	0.878

**TABLE 3:** Imaging parameters for WHIRLED PEAS sequences.

Parameter	*2D hybrid SE/GRE*	*3D single‐echo GRE*	*3D multi‐echo GRE*	
FOV/Resolution (cm/mm)	24 × 24/0.8 × 0.8	24 × 24 1 × 1	30 × 30/2 × 2	30 × 30/2 × 2
No. of slices/slice thickness (mm)	210/0.8	160/1	100/2	100/2
k‐space trajectory	Spiral in‐out (LQE^17^)	Spiral out (spiral staircase^18^)	Spiral in‐out‐in‐out (spiral staircase^18^)	Spiral out‐in‐out‐in (spiral staircase^18^)
τ (ms)	20	4	2	2
$\bar{G}$ (mT/m)	40	39.75	27.71	27.71
$\bar{S}$ or ${\bar{S}}_{⊥}$ (mT/m/ms)	120	120	120	120
$\bar{f}$ (kHz)	1	1	1	1
Number of arms	16	56	60	60
TE/TR (ms)	100/3000	1/9.6	6.93 (TE1), 10.96 (TE2)/16.7	0.99 (TE1), 5.0 (TE2), 9.01 (TE3)/13.6
Flip angle ( 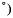	180	12	10	10
Arm ordering	Mixed	Mixed	Linear	Linear

Abbreviations: SE = spin‐echo, GRE = gradient echo, LQE = Localized Quadratic Encoding.

### 4.2 Slew constraint

In Table 4, the first WHIRLED PEAS column $S_{∥}$ gives the value of the slew parallel to *G*, which jumps to half its value between the $\bar{\omega }$‐constrained and $S_{⊥}$‐constrained segment. The biggest discontinuity is in use case 2, where the jump is 3.5% the maximum slew constraint of 150 mT/m/ms. Also in Table 4, the maximum value of slew magnitude S is given, to compare it with the slew constraint, which is only applied to $S_{⊥}$. The biggest discrepancy is in use case 2, for which the total slew rate is 0.25% greater than the maximum limit of 150 mT/m/ms.

### 4.3 Software and scanner implementation

**FIGURE 3:**
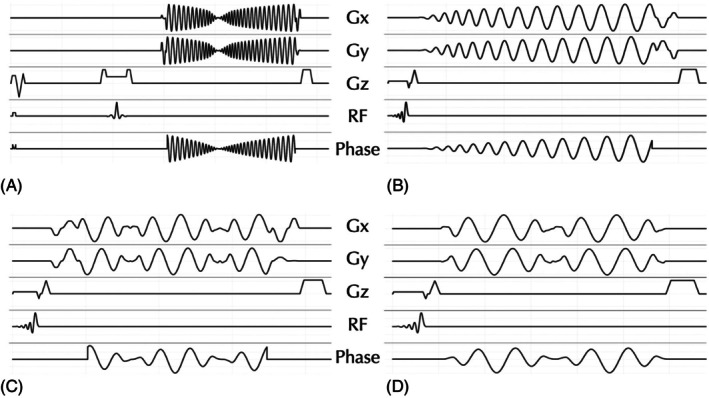
Examples of WHIRLED PEAS implementations using modular waveforms. (A) shows a 2D spin‐echo sequence, whereas (B)–(D) depict 3D gradient‐echo sequences. (A) A T2W spin‐echo with a spiral in‐out trajectory to maximize data acquisition efficiency. (B) A standard T1w 3D spiral‐out sequence. (C) and (D) illustrate 3D multi‐echo spiral trajectories with in‐out‐in‐out and out‐in‐out‐in patterns, respectively. Phase waveforms were computed using analytical k‐space coordinates and applied prospectively for FOV correction. These variations highlight WHIRLED PEAS' flexibility for spin‐echo and gradient‐echo imaging. T2W = T2‐weighted, T1W = T1‐weighted.

**FIGURE 4:**
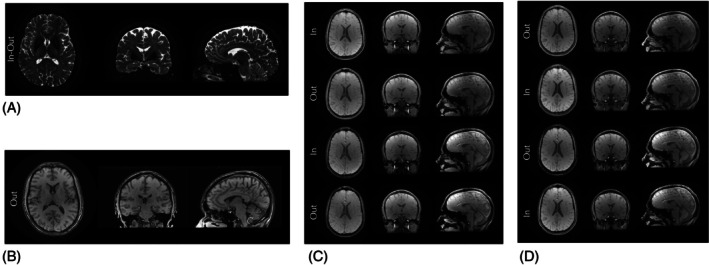
Example images acquired using different implementations of the WHIRLED PEAS sequence. (A) presents images from a 2D T2W spin‐echo sequence using a spiral in‐out trajectory, as illustrated in Figure 3A. (B) displays images acquired using a 3D single‐echo gradient‐echo sequence with a standard spiral‐out trajectory, as illustrated in Figure 3B. (C) and (D) show images from a 3D multi‐echo gradient‐echo sequence employing an in‐out‐in‐out and out‐in‐out‐in trajectory respectively, as illustrated in Figure 3C,D.

Figures 3 and 4 illustrate different implementations of WHIRLED PEAS and their corresponding imaging results, demonstrating its feasibility for both simulation and scanner execution. The results highlight the flexibility of WHIRLED PEAS in optimizing spin‐echo and gradient‐echo acquisitions while ensuring efficient trajectory generation and scanner compatibility.

## 5 DISCUSSION

The results of Table 1 demonstrate that the performance of WHIRLED PEAS is strikingly similar to that of a numerically optimized Archimedean spiral, both in terms of acquisition time t and relative SNR W. This confirms that the proposed analytical equations provide an accurate and efficient alternative to traditional numerical trajectory design method. Whereas the slew constraint differs slightly between the two methods, the impact on trajectory fidelity and imaging performance is negligible in most practical scenarios.

One of the advantages of WHIRLED PEAS is its nimble scanner implementation, which allows full k‐space coordinates and SDC weights to be precomputed during protocol optimization and directly passed to the reconstruction pipeline. This eliminates the need for traditional retrospective k‐space computation, reducing processing overhead and simplifying reconstruction. Additionally, the computed k‐space coordinates can be used to support prospective FOV off‐center shift phase correction during acquisition—a feature available on certain vendor platforms—and WHIRLED PEAS is well suited to take advantage of it due to the availability of precise, analytical k‐space coordinates. This capability ensures accurate spatial encoding without the need to increase FOV to compensate for shift‐related blurring, improving acquisition efficiency. The fully analytical nature of WHIRLED PEAS also makes it highly computationally efficient, allowing direct computation of gradient waveforms, k‐space trajectories, and density correction weights without iterative numerical solvers. This efficiency makes WHIRLED PEAS well suited for real‐time applications, including adaptive imaging sequences that dynamically adjust trajectories.

The modular nature of WHIRLED PEAS enables flexible trajectory designs, including spiral‐out, spiral‐in, in‐out, out‐in, and multi‐echo spiral sequences, making it an attractive option for various applications such as multi‐echo imaging, T2‐weighted imaging, and dynamic MRI. Additionally, WHIRLED PEAS provides a structured approach for handling off‐resonance effects and motion artifacts. The analytical equations directly map acquisition time as a function of k‐space position, which is critical for off‐resonance correction and deblurring techniques. Furthermore, generating smooth, moment‐nulled trajectories enhances robustness against motion‐induced phase errors.

Our group is exploring the expansion of WHIRLED PEAS to include variable density sampling, although, in practice, we find that uniform undersampling has better blurring properties. This is because, for a fixed *τ*, preferentially undersampling the outer parts of k‐space results in a slower radial speed through k‐space in the center, where the majority of signal exists. It is this radial speed through k‐space that determines the extent of blurring. A future comparison of variable and uniform density sampling is warranted to better understand these tradeoffs; however, our current efforts remain with the common trajectory discussed here, combined with undersampling through use of a reduced set of equally space arms. The analytic nature of the waveforms speeds up computation, improves analysis, and enables more nimble pulse sequence development.

## 6 CONCLUSION

WHIRLED PEAS provides an exact, time‐dependent analytical framework for generating spiral trajectories and their corresponding gradient waveforms, k‐space coordinates, SDC, and k‐space time maps. Unlike conventional numerical methods, WHIRLED PEAS eliminates the need for iterative optimization, offering a computationally efficient and easy‐to‐implement solution that maintains performance comparable to numerically derived spiral trajectories.

A key advantage of WHIRLED PEAS is its nimble scanner implementation, where precomputed k‐space coordinates and SDC weights are directly passed to the reconstruction pipeline, eliminating the need for retrospective k‐space computation. This combination of analytical precision, computational efficiency, and real‐time adaptability makes WHIRLED PEAS an attractive choice for modern spiral MRI applications.

Beyond its current applications, WHIRLED PEAS offers a modular and flexible design, supporting various trajectory configurations, including spiral‐in, spiral‐out, spiral‐in‐out, spiral‐out‐in, and multi‐echo sequences. Overall, WHIRLED PEAS represents a powerful, scalable, and analytically rigorous approach to spiral trajectory design, providing a practical and efficient alternative to numerical methods.

## Data Availability

The complete software implementation, as archived at the time of publication—including the GPI node for simulation and the C++ modules—is available in WHIRLEDPEAS.zip (attached to this release). Additionally, the WHIRLEDPEAS node will be included in GPI Core Node Library version 2.3.3 and later. It can be installed via Conda from the Conda‐Forge channel or cloned as source code from: SourceCodeForCoreNodes

## References

[1] Ahn CB , Kim JH , Cho ZH . High‐speed spiral‐scan echo planar NMR imaging‐I. IEEE Trans Med Imaging. 1986;5:2‐7. 18243976 10.1109/TMI.1986.4307732

[2] Meyer CH , Hu BS , Nishimura DG , Macovski A . Fast spiral coronary artery imaging. Magn Reson Med. 1992;28:202‐213. 1461123 10.1002/mrm.1910280204

[3] Glover GH . Simple analytic spiral K‐space algorithm. Magn Reson Med. 1999;42:412‐415. 10440968 10.1002/(sici)1522-2594(199908)42:2<412::aid-mrm25>3.0.co;2-u

[4] Salustri C , Yang Y , Glover GH . Simple but reliable solutions for spiral MRI gradient design. J Magn Reson. 1999;140:347‐550. 10497042 10.1006/jmre.1999.1831

[5] Kim DH , Adalsteinsson E , Spielman DM . Simple analytic variable density spiral design. Magn Reson Med. 2003;50:214‐219. 12815699 10.1002/mrm.10493

[6] Pipe JG , Zwart NR . Spiral trajectory design: a flexible numerical algorithm and base analytical equations. Magn Reson Med. 2014;71:278‐285. 23440770 10.1002/mrm.24675

[7] Pipe JG , Borup DD . Generating spiral gradient waveforms with a compact frequency spectrum. Magn Reson Med. 2022;87:791‐799. 34519379 10.1002/mrm.28993

[8] Pipe JG . An optimized center‐out k‐space trajectory for multishot MRI: comparison with spiral and projection reconstruction. Magn Reson Med. 1999;42:714‐720. 10502760 10.1002/(sici)1522-2594(199910)42:4<714::aid-mrm13>3.0.co;2-g

[9] Barbero S , Ritoré M . Circle involute as an optimal scan path, minimizing acquisition time, in surface topography. Surf Topogr Metrol Prop. 2019;7:35010.

[10] Oberhammer T , Weiger M , Hennel F . Spiral MRI trajectory design with frequency constraint. In Proceedings of the 18th Annual Meeting of ISMRM, Stockholm, Sweden, 2010. p. 4969.

[11] Pipe JG . WHIRLED PEAS: analytical equations for spiral trajectories and matching gradient waveforms. In Proceedings of the Joint ISMRM & ISMRT Annual Meeting, Toronto, Ontario, Canada, 2023. p. 2378.

[12] Pipe JG . Expanding WHIRLED PEAS: extensions, analysis, and code for new analytical waveforms for spiral MRI. In ISMRM Workshop on Data Sampling & Image Reconstruction Sedona, AZ, 2023. p. 63.

[13] Wang D , Zwart NR , Pipe JG . Joint water‐fat separation and deblurring for spiral imaging. Magn Reson Med. 2018;79:3218‐3228. 28983966 10.1002/mrm.26950

[14] Johnson KO , Pipe JG . Convolution kernel design and efficient algorithm for sampling density correction. Magn Reson Med. 2009;61:439‐447. 19165893 10.1002/mrm.21840

[15] Zwart NR , Pipe JG . Graphical programming interface: a development environment for MRI methods. Magn Reson Med. 2015;74:1449‐1460. 25385670 10.1002/mrm.25528

[16] Pipe JG , Ahunbay E , Menon P . Effects of interleaf order for spiral MRI of dynamic processes. Magn Reson Med. 1999;41:417‐422. 10080293 10.1002/(sici)1522-2594(199902)41:2<417::aid-mrm29>3.0.co;2-w

[17] Kim D , Wang D , Chao T , Campeau N , Pipe JG . Volumetric T_2_‐weighted spin echo imaging with improved SNR using localized quadratic encoding and a spiral readout trajectory. Magn Reson Med. 2023;90:2261‐2274. 37639386 10.1002/mrm.29788

[18] Anderson AG III , Wang D , Pipe JG . Controlled aliasing for improved parallel imaging with a 3D spiral staircase trajectory. Magn Reson Med. 2020;84:866‐872. 31967342 10.1002/mrm.28154

